# Whispered Struggles in the Workplace: A Scoping Review of Cognitive and Interpersonal Challenges and Organizational Strategies for Employees living with Dementia

**DOI:** 10.1002/alz.085684

**Published:** 2025-01-09

**Authors:** Shruti Jindal, Sabrina Pit, Mohammad Hamiduzzaman

**Affiliations:** ^1^ Jamia Millia Islamia, New Delhi, New Delhi India; ^2^ University of Sydney, Camperdown, NSW Australia

## Abstract

**Background:**

Dementia, including its younger‐onset variant, increasingly challenges diverse workplaces, affecting both employees living with dementia and their employers. With a growing global trend of individuals extending their working years, it becomes crucial to understand the specific challenges and implications for those with dementia within various employment contexts. This review delves into the cognitive, interpersonal, and organizational challenges faced by employees living with dementia across a broad range of workplaces, reflecting the evolving dynamics of today’s work environment.

**Method:**

In this scoping review, we employed Arksey and O’Malley’s framework, using a structured approach to explore the intersection of dementia and employment. Our methodology, shaped by the PRISMA‐P guidelines, involved a systematic search in PubMed and PsycINFO for studies published from 2000 onwards. We utilized a comprehensive set of search terms and Boolean operators, focusing on the experiences and challenges faced by employees living with dementia. This included assessing workplace adjustments, organizational support strategies, and employee well‐being. The selection criteria were defined to include studies that specifically addressed cognitive and interpersonal challenges in the workplace, avoiding broader dementia‐related topics to maintain focus on our research objectives.

**Result:**

The review systematically identified key areas of concern and gaps in the current literature regarding dementia in the workplace. It underlined the necessity for workplace adjustments, including flexible hours and modified job responsibilities, to support employees with dementia. The role of organizations in facilitating appropriate workplace accommodations and support strategies, such as supportive supervision and access to tailored occupational health services, was also a significant finding. More importantly, this scoping review identified critical questions for future investigation, including the efficacy of current workplace adjustments, the role of policy in shaping organizational support, and the long‐term impact of such accommodations on both the employee with dementia and workplace dynamics.

**Conclusion:**

This review underscores the need for comprehensive workplace strategies and policy reforms to support employees with dementia, highlighting the critical role of organizational support and flexibility.

